# Long-term outcome of catheter ablation and other form of therapy for electrical storm in patients with implantable cardioverter-defibrillators

**DOI:** 10.1007/s10840-017-0291-1

**Published:** 2017-10-24

**Authors:** Stanislaw Morawski, Patrycja Pruszkowska, Beata Sredniawa, Radoslaw Lenarczyk, Zbigniew Kalarus

**Affiliations:** 1 Department of Cardiology, Congenital Heart Diseases and Electrotherapy, School of Medicine with Division of Dentistry in Zabrze, Medical University of Silesia, M.C Sklodowskiej Street 9, 40-055 Zabrze, Poland; 2 Silesian Center for Heart Diseases, M.C Sklodowskiej Street 9, 40-055 Zabrze, Poland

**Keywords:** Electrical storm, Catheter ablation, Ventricular tachycardia, Ventricular fibrillation, Implantable cardioverter-defibrillator

## Abstract

**Purpose:**

Radiofrequency catheter ablation (RFCA) for electrical storm (ES) has become a widely used therapeutic method. Its effectiveness in comparison to other forms of ES treatment is however uncertain.

**Methods:**

This single-centre retrospective study investigated the long-term clinical outcome after RFCA for ES and compared long-time effects of ablation to other forms of treatment. The study population consisted of 70 consecutive patients hospitalised between January 2010 and June 2015 due to ES. Patients were recruited for the study if the following criteria were fulfilled: first ES caused by ventricular tachycardia (VT) or ventricular fibrillation (VF), implanted cardioverter defibrillator or cardiac resynchronisation therapy device and left ventricular ejection fraction < 50%. The follow-up data on VT/ES recurrence was obtained from pacemaker/implanted cardioverter defibrillator memory. Data on all-cause mortality was collected during outpatient visits or by telephone contact.

**Results:**

Of the 70 patients enrolled, 28 (40%) were treated with RFCA (group A) and 42 (60%) received other forms of treatment for ES (group B). During a mean (±SD) 864 (629) days of follow-up, death occurred in 4 (14.3%) patients in the ablation group and in 16 (38.1%) patients treated with other methods [*p* = 0.03]. There was no significant between-group difference in VT/VF and ES recurrence. Statistical analysis revealed that the presence of cardiac resynchronisation therapy device during ES, stroke and/or transient ischaemic attack and lower baseline hematocrit level were the multivariate predictors of all-cause mortality.

**Conclusions:**

In patients treated with RFCA for ES, all-cause mortality was significantly lower compared to the group treated with other methods.

**Electronic supplementary material:**

The online version of this article (10.1007/s10840-017-0291-1) contains supplementary material, which is available to authorized users.

## Introduction

Heart failure (HF) is a growing medical problem in developed countries, with incidences among adult population reaching up to 2% [1]. It is well known that HF patients are simultaneously at a significantly higher risk of sudden cardiac death. The recommended and most efficient form of primary and secondary prevention against sudden cardiac death in heart failure patients is the implantation of automatic implantable cardioverter defibrillators (ICD) [2, 3]. 3.5% of patients with an ICD for primary prevention, and even 10–40% of those implanted for secondary prevention of SCD, will suffer from the most malignant form of ventricular arrhythmia, known as an electrical storm (ES) [4, 5]. An electrical storm is defined as the occurrence of three or more distinct episodes of ventricular tachycardia (VT) and/or ventricular fibrillation (VF) within 24 h [6]. In patients with ICD, its usual presentation involves multiple antitachycardia pacing therapies (ATP) and/or high-energy therapies within a short-time period. Management in this challenging group is generally focused on the elimination of potentially reversible causes of electrical instability, haemodynamic stabilisation and (once reversible causes have been excluded or dampened) modification of the arrhythmogenic substrate with catheter ablation of ventricular tachycardia [7–9]. Most of the published data on radiofrequency catheter ablation (RFCA) for ES is based on small series or case reports, mainly describing single treatment method. No large studies have compared RFCA with conservative treatment and their effects on survival. The purpose of this study was to assess the long-term clinical outcomes after RFCA for ES and to compare this method with other forms of treatment for ES.

## Methods

### Patients and eligibility criteria

The study population consisted of 70 consecutive patients, who were hospitalised between January 2010 and June 2015 due to the occurrence of an electrical storm. In 68 patients, the arrhythmia responsible for the occurrence of ES was VT; in 2, the ES was due to VF.

Patients were initially recruited for the study if they fulfilled the following criteria: diagnosed with the first electrical storm caused by VT or VF, previous or present implantation of an ICD or cardiac resynchronisation therapy device (CRT-D) during the first ES hospitalisation, and heart failure with left ventricular ejection fraction (LVEF) < 50% (assessed by transthoracic echocardiography). Patients were excluded if they were suffering from inherited arrhythmogenic diseases (long QT syndrome, Brugada syndrome, catecholaminergic polymorphic ventricular tachycardia, short QT syndrome arrhythmogenic right ventricular cardiomyopathy), had suffered from a previous electrical storm, had been previously treated with VT ablation or were aged < 18 years old. The clinical characteristics of the total study population of patients with a particular emphasis on age, sex, comorbidity, basic electrocardiographic, echocardiographic parameters, the type of cardiomyopathy and the type of prevention causing ICD/CRT-D implantation are shown in Table 1. To evaluate the effect of catheter ablation for the long-term prognosis of patients treated because of an electrical storm, the research group was divided depending on ablation procedure (group A) or no ablation procedure (group B) during primary hospitalisation. The clinical characteristics of group A (28 patients) were compared with those of the remaining study population (42 patients; group B) as show in Table 1.

**Table 1:** Baseline clinical and demographic characteristics of the study population, ablated (group A) and no ablated (group B) group

Group characteristics	*N* = 70	Group A (*n* = 28)	Group B (*n* = 42)	*p*
Gender (M/F)	64/6	26/2	38/4	0.73
Age	64.71 ± 10.71	65.75 ± 8.93	64.02 ± 11.8	0.51
BMI (kg/m^2^)	27.59 ± 3.72	28.66 ± 3.65	26.76 ± 3.61	0.04
NYHA class	2.16 ± 0.69	2.16 ± 0.62	2.17 ± 0.75	0.97
NYHA ≥ 2	57 (81.4%)	24 (85.7%)	33 (78.6%)	0.46
QRS duration (ms)	154 ± 39	159.71 ± 40.86	150.23 ± 38.15	0.33
Native QRS duration (ms)^a^	130.59 ± 28.33	140.61 ± 30.42	122.0 ± 23.88	0.04
Paced QRS duration (ms)^b^	183.52 ± 30.21	194.1 ± 34.9	178.48 ± 27.17	0.18
Heart rate/min	68.14 ± 11.44	66.96 ± 10.79	69.93 ± 11.92	0.49
Ventricular stimulation	31 (44.3%)	10 (35.7%)	21 (50%)	0.24
LVEF (%)	26.2 ± 7.31	27.11 ± 6.27	25.59 ± 7.95	0.40
LVEDD (mm)	67.91 ± 8.33	67.73 ± 8.15	68.03 ± 8.55	0.89
LVESD (mm)	55.78 ± 11.23	53.75 ± 10.86	57.2 ± 11.42	0.25
EDV (ml)	237.96 ± 78.26	233.75 ± 80.99	241.19 ± 77.53	0.75
ESV (ml)	175.63 ± 74.51	164.93 ± 69.65	182.76 ± 78.44	0.49
MVR-severe	2 (2.9%)	0 (0%)	2 (4.8%)	0.24
LA (mm)	45.25 ± 6.82	46.52 ± 6.94	44.45 ± 6.72	0.26
Implantation of ICD/CRTD in secondary prevention	42 (60%)	19 (67.9%)	23 (54.8%)	0.28
Secondary prevention due to VF/SCA	19 (27.1%)	9 (32.1%)	10 (23.8%)	0.45
During the storm, the presence of an implanted CRT-D	24 (34.3%)	8 (28.6%)	16 (38.1%)	0.42
During the storm, the presence of an implanted ICD	41 (58.6%)	18 (63.3%)	23 (54.8%)	0.44
Ischemic cardiomyopathy	57 (81.4%)	25 (89.3%)	32 (76.2%)	0.17
MI	54 (77.1%)	23 (82.1%)	31 (73.8%)	0.42
Previous myocardial infarction treated conservatively	39 (55.7%)	20 (71.4%)	19 (45.2%)	0.03
Number of MI	1.23 ± 1.04	1.18 ± 0.86	1.26 ± 1.14	0.74
Angioplasty other than MI	33 (47.1%)	14 (50%)	19 (45.2%)	0.70
Coronary artery bypass grafting	13 (18.6%)	8 (28.6%)	5 (11.9%)	0.08
Complete revascularisation after angiography	43 (61.4%)	19 (67.9%)	24 (57.1%)	0.36
Arterial hypertension	44 (62.9%)	16 (57.1%)	28 (66.7%)	0.42
Diabetes type 2	25 (35.7%)	12 (42.9%)	13 (31%)	0.32
Chronic kidney disease	26 (37.1%)	8 (28.6%)	18 (42.9%)	0.23
Stroke/transient ischaemic attack	12 (17.1%)	4 (14.3%)	8 (19%)	0.61
Chronic obstructive pulmonary disease	7 (10%)	2 (7.1%)	5 (11.9%)	0.52
Atrial fibrillation	31 (44.3%)	13 (46.4%)	18 (42.9%)	0.77
Hyperthyroidism	8 (11.4%)	4 (14.3%)	4 (9.5%)	0.55
Amiodarone	32 (45.7%)	10 (35.7%)	22 (52.4%)	0.18
Angiotensin-converting-enzyme inhibitors	60 (85.7%)	24 (85.7%)	36 (85.7%)	1.0
Beta-blockers	70 (100%)	28 (100%)	42 (100%)	1.0
Class I antiarrhythmic agents	3 (4.3%)	2 (7.1%)	1 (2.4%)	0.34
Mineralocorticoid receptor antagonist	60 (85.7%)	25 (89.3%)	35 (83.3%)	0.49
Loop diuretics	52 (74.3%)	21 (75%)	31 (73.8%)	0.91
Thiazides	3 (4.3%)	1 (3.6%)	2 (4.8%)	0.81
Digitalis	8 (11.4%)	3 (10.7%)	5 (11.9%)	0.88
Lipid-lowering statin drugs	62 (88.6%)	26 (92.9%)	36 (85.7%)	0.36
Insulin	7 (10%)	4 (14.3%)	3 (7.1%)	0.34
Oral antidiabetic drugs	13 (18.6%)	7 (25%)	6 (14.3%)	0.27
Sedatives	0			
Anaesthetics	0			

*BMI* body mass index, *CRT-D* cardiac resynchronization therapy defibrillator, *EDV* end-diastolic volume, *ESV* end-systolic volume, *ICD* implantable cardioverter-defibrillator, *LA* left atrium, *LVEDD* left ventricular end-diastolic diameter, *LVEF* left ventricular ejection fraction, *LVESD* left ventricular end-systolic diameter, *MI* myocardial infarction, *MVR* mitral valve regurgitation, *NYHA class* New York Heart Association Class, *SCA* sudden cardiac arrest, *VF* ventricular fibrillation

^a^Native QRS duration—duration of QRS complex in patients without ventricular stimulation

^b^Paced QRS duration—duration of QRS complex in patients with ventricular stimulation

During hospitalisation, if presenting VT episodes were recorded based on 12-lead ECG and/or 12-lead Holter ECG, the corresponding ECG morphologies were defined as clinical VT. Coronary angiography was performed in 44 patients in whom myocardial ischaemia was suspected. In the remaining population, coronary arteriography was performed in referral hospitals (5 patients) and in the previous 12 months (9 patients).

In 7 patients, this was not conducted because of no clinical suspicion of myocardial ischaemia or because of non-ischaemic cardiomyopathy (5 patients). Percutaneous coronary intervention was performed in 18 patients.

No additional non-pharmacological beta blockade procedures were performed (such as left stellate ganglion blockade). No overdrive atrial pacing was used to tide over the storm in any of the patients. Patients underwent standard treatment in terms for reversible reasons of ES. In retrospective analysis during primary hospitalisation, 28 patients were treated using the catheter ablation. A RFCA was performed in all patients who underwent the invasive electrophysiological study.

### Electrophysiological study and ablation strategy

Written informed consent was obtained from all patients before RFCA. All procedures were performed under local anaesthesia. In all cases, retrograde femoral access was used. Mapping was performed using the three-dimensional electro-anatomical mapping system Carto 3 (Biosense Webster, Diamond Bar, CA, USA) and a saline irrigated tip catheter NaviStar ThermoCool (Biosense Webster). In all cases, endocardial mapping was routinely undertaken. An initial bolus of heparin, in dose 80 IU/kg of patient body weight, was administered after the electrode was positioned in the left ventricle. Every 15 min, the activating clothing time (ACT) was measured and an additional heparin bolus was administered to maintain ACT above 250 s. In all patients in sinus rhythm (SR), three-dimensional left ventricular bipolar voltage maps were constructed. A dense scar was defined as an area of local bipolar peak-to-peak voltage of < 0.5 mV and a border zone < 1.5 mV. In patients with haemodynamically stable VT, arrhythmia was induced by programmed stimulation and a three-dimensional activating map was obtained as in patients with an incessant VT or if arrhythmia occurred spontaneously.

Targets for ablation were determined as mid-diastolic potentials, areas of slow conductions, or identifying isthmus and exit zones with prolonging time stimulus to QRS in sinus rhythm (SR) patients using pacemapping. During VT, the target for application was determined in areas central to the exit zone and identified by entrainment. Radiofrequency current was delivered with an open irrigated tip catheter with a 30 ml/min saline flow at a power setting of 30 up to 50 W and a temperature limit of 45 °C. After ablation, programmed stimulation with up to three extra stimuli was performed. The success was defined as non-inducibility of clinical VT. In cases of inducibility, another form of sustained VT, additional mapping and ablation was performed according to the protocol described above. Apart from intra-procedural anticoagulation with unfractionated heparin, in every patient, a standard prophylactic dose of low-molecular-weight heparin was administered in the early post-procedural period. Further anticoagulation was carried out with the use of vitamin K antagonists, which were instituted on the first post-operative day, and continued for 1 month, with the target INR between 2 and 3. This therapy was subsequently withdrawn, if long-term anticoagulation was not required due to other reasons.

### Follow-up

The majority of the patients (65; 92.9%) already had implanted ICD (41; 58.6%) or CRTD (24; 34.3%) before primary admission. In the rest, ICD (5; 7.1%)/CRDT (0; 0%) devices were implanted before the discharge.

The follow-up date for VT/ES recurrence/mortality (average 864 days; mean 706 days, SD 629) was obtained in our pacemaker/ICD outpatients’ clinic through interrogation of the devices, remote monitoring (RM), subsequent hospitalisations and telephone contact.

### Statistical analysis

Continuous variables are described as mean ± standard deviation (SD), or median and range for skewed distributions. Categorical variables are described as numbers and percentages. Between-group comparisons were performed with Student’s *t* test or the *χ* ^2^ test as appropriate. Univariate and multivariate analyses were performed using a Cox proportional hazards model, with hazard ratio (HR) and 95% confidence intervals (CI) reported. Two multivariate Cox regression models were constructed to predict long-term mortality. The first tested parameters which had significance in the univariate model < 0.05 (model 1), and the second included variables with *p* < 0.1 (model 2). Some variables (e.g. creatinine level) were not included into multivariate models due to redundancy with other parameters already included (chronic kidney disease in this case).

Long-term success (i.e. time to VT recurrence, time to ES recurrence and time to death) was also examined in a survival analysis framework. Time-to-event survival curves were graphed for different patient subgroups using the Kaplan–Meier estimation technique. Differences between strata were assessed by a log-rank test.

The study was carried out in accordance with the Declaration of Helsinki and International Committee on Harmonization guideline for good clinical practice.

## Results

Seventy consecutive patients (91.4% male, age [64.71 ± 10.71] years; LVEF 26.2% ± 7.31%), the majority with ischaemic cardiomyopathy (81.43%) and ES, constituted our study population. As described in the “Methods” section, patients were divided into two groups according to execution of catheter ablation during the primary episode of ES. Among the groups, no significant difference in baseline clinical and electrocardiography characteristics were observed except BMI (group A: 28.66 ± 3.65 vs. group B: 26.76 ± 3.61; *p* = 0.04), myocardial infarction treated non-invasively (20 [71.4%] vs. 19 [45.2%]; *p* = 0.03) and duration of native QRS respectively (group A: 140.61 ± 30.42 ms vs. group B: 122.0 ± 23.88 ms; *p* = 0.04) (Table 1).

**Fig. 1:**
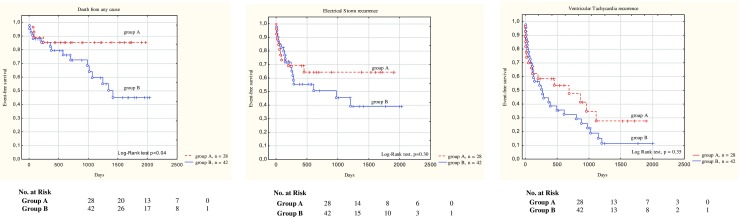
Kaplan–Meier event-free survival estimates in the patient population during follow-up (group A vs. group B). The Kaplan–Meier curve shows a difference between group A (RFCA treatment in primary hospitalisation) and group B (no-RFCA in primary hospitalisation) in terms of total mortality, electrical storm recurrence and ventricular tachycardia recurrence at 864 ± 629 days of follow-up. A statistically significant difference in terms of total mortality is observed (log-rank test, *p* = 0.04)

In group A, the majority of cases showed no clear cause of ES during primary hospitalisation (*n* = 24 [85.71%]), in contrast to group B, where nearly half of the patients had clear causes of ES (*n* = 22 [53.4%]). In group B, RFCA was abandoned in 20 patients during hospitalisation despite undetected and potentially reversible cause of electrical instability, due to the disappearance of arrhythmia as a result of the intensification of antiarrhythmic therapy (increased doses of β-blockers (20 patients) and to the inclusion of treatment with antiarrhythmic class III drugs (14 patients; 13 with amiodarone, 1 with sotalol) (Table 2). After the first ES, 28 patients underwent a catheter ablation procedure. Nine (32.1%) patients from group A and 13 (31%) from B group underwent subsequent ablation during the follow-up because of VT and ES recurrence (*p* = 0.92). Importantly, no statistical differences were observed in terms of VT (group A: 16 [57.1%]; group B: 31 [73.8%]; *p* = 0.15) and ES (group A: 9 [32.1%]; group B: 19 [45.2%]; *p* = 0.28) recurrence in both groups. However, there was significantly lower all-cause mortality in group A (4 [14.3%] vs. 16 [38.1%]; *p* = 0.03) (Table 3). Significant differences in survival between ablated and non-ablated groups are shown in Fig. 1. At the end of the ablation procedures, 23 patients underwent programmed electrical stimulation. In 2 of those patients, non-clinical VTs were inducible. There was no inducible clinical VT at the end of the procedure. Five patients (17.9%) were not tested due to haemodynamic instability after CA.

**Table 2:** Primary episode of ES—causes

	Group A (*n* = 28)	Group B (*n* = 42)	*p*
No potentially reversible cause	24 (85.7%)	20 (47.6%)	<0.01
Acute coronary syndrome	1 (3.6%)	5 (11.9%)	0.23
Significant stenosis of the coronary artery requiring intervention	4 (14.3%)	16 (38.1%)	0.03
Infective endocarditis	0 (0%)	3 (7.1%)	0.15
Dyselectrolytemia	0 (0%)	2 (4.8%)	0.25
Discontinuation of medication	0 (0%)	1 (2.4%)	0.42
Exacerbation of heart failure	0 (0%)	1 (2.4%)	0.42
Decompensated hyperthyroidism	0 (0%)	1 (2.4%)	0.42
More than 1 cause	1 (3.6%)	7 (16.7%)	0.09

**Table 3:** Long-term treatment effect of ES in study population, ablated (group A) and no ablated (group B) group

	*N* = 70	Group A (*n* = 28)	Group B (*n* = 42)	*p*
Ventricular tachycardia/ventricular fibrillation recurrence	47 (67.1%)	16 (57.1%)	31 (73.8%)	0.15
Recurrence of an electrical storm	28 (40%)	9 (32.1%)	19 (45.2%)	0.28
Number of subsequent electrical storms	0.8 ± 1.48	0.43 ± 0.69	1.05 ± 1.79	0.09
In-hospital death	4 (5.7%)	1 (3.6%)	3 (7.1%)	0.54
All-cause death	20 (28.6%)	4 (14.3%)	16 (38.1%)	0.03
Organ heart transplantation	1 (1.4%)	1 (3.6%)	0 (0%)	0.22
Composite of all-cause death, electrical storm recurrence and ventricular tachycardia/ventricular fibrillation recurrence	57 (81.4%)	21 (75%)	36 (85.7%)	0.27

Analysis of the early post-operative period (< 30 days) revealed a non-significantly (*p* = 0.16) higher rate of VA recurrence rates in the group which underwent RFCA (7 [25%]) vs. the conservative group (5 [12%]), respectively. However, sub-analysis of the late (> 30 days postoperatively) period only, a significantly (*p* = 0.02) lower rate of recurrences was found in the ablated group (46.4%) vs. in the non-ablated group (75%; 2 patients from the non-ablated group died during the blanking period, and consequently, they were not included in the analysis).

Variables evaluated in the univariate analysis were as follows: age, gender, BMI, NYHA class, LVEF, severe MVR, implantation of ICD/CRTD in secondary prevention of SCA, presence of an implanted CRT-D (during the storm), ischemic cardiomyopathy, previous myocardial infarction treated conservatively, complete revascularisation after angiography, diabetes type 2, chronic kidney disease, stroke and/or transient ischaemic attack, chronic obstructive pulmonary disease, atrial fibrillation, haematocrit level, C-reactive protein level, creatinine level, GFR, NT-pro-BNP level, potentially reversible causes of ES, primary RFCA procedure, VT recurrence and ES recurrence.

The univariate analysis revealed that severe mitral valve regurgitation (MVR), presence of CRT-D during ES, diabetes type 2, stroke and/or a transient ischaemic attack, a lower baseline haematocrit level, a higher NT-pro-BNP level, a higher C-reactive protein (CRP) level and infective endocarditis as a potentially reversible cause of ES were significant prognostic predictors for all-cause mortality. Further details are provided in Table S1 in the Supplementary Appendix.

The multivariate Cox regression model, incorporating parameters which had significance in the univariate model < 0.05 as covariates (model 1), demonstrated that both the presence of CRT-D during ES and stroke and/or a transient ischaemic attack were independently associated with the increased risk of total mortality (HR 7.99, 95%CI 2.35–27.16; *p* = 0.001; and HR 4.78, 95%CI 1.43–15.93; *p* = 0.01, respectively). In the second model (including all variables with *p* < 0.1), the presence of CRT-D during ES (HR 6.83, 95%CI 1.93–24.24; *p* = 0.003), stroke and/or a transient ischaemic attack (HR 9.86, 95%CI 1.71–56.65; *p* = 0.01) and a lower baseline haematocrit level (HR 0.84, 95%CI 0.72–0.99; *p* = 0.04) were all independently associated with the higher risk of all-cause mortality. A thorough analysis of these multivariates analyses (both models 1 and 2) is also provided in Table S1 in the Supplementary Appendix.

## Discussion

Recurrent ventricular arrhythmias, which as they are the cause of electrical storms, are the most common problem among ICD/CRT-D recipients. In our study, catheter ablation in such patients was more effective than any other form of therapy in reducing death at any time. Despite the interdisciplinary approach to the treatment, long-term mortality remains high. In our study, more than a quarter of patients died during the course of the study, with the majority of deaths taking place during a few months after an episode of electrical storm. This is concordant with several studies, which have shown that ES occurrence in patients with ICD/CRT-D is an independent risk factor for death, with an increase in risk of at least 2–3-fold in long-term follow-up. The risk is highest in the first 3 months post an electrical storm episode [5, 10–14]. For many years, consensus statements and guidelines have recommended the use of catheter ablation (CA) when antiarrhythmic therapy does not prevent recurrences of ventricular arrhythmias; however, these recommendations have been largely based on expert opinion and non-randomised case series [15–17]. These recommendations were in force up until the publication of the 2015 ESC guidelines for the management of patients with ventricular arrhythmias and the prevention of sudden cardiac death where urgent catheter ablation is recommended (classification of recommendation II B) in patients with scar-related heart disease with incessant VT or electrical storm [18]. However, these guidelines were mainly created based on the Carbucicchio et al. study, where catheter ablation for the management of ES was evaluated in detail [9]. The authors included 95 patients with structural heart disease and electrical storm refractory to antiarrhythmic drug therapy. At a median follow-up of 22 months, ES recurred in 8% and cardiac mortality was significantly higher in patients in whom at least one clinical VT could not be abolished, when compared with patients after successful CA defined as non-inducible VT in programmed electrical stimulation. Similar findings were reported by Nayyar et al. [14] in their meta-analysis of 39 publications including a total number of 471 patients with structural and non-structural heart disease, who underwent invasive ablation management of ventricular arrhythmia (VA) storm with total follow-up 61 ± 37 weeks. The odds of death were found to be four times higher after a failed procedure, compared to those with a successful procedure (95%CI 2.04–8.01; *p* < 0.001). The value of information from our study is that it focuses on patients with structural heart disease and with a first episode of ES, excluding those who had already undergone previous RFCA. Our trial provides evidence that catheter ablation is the most effective first-line life-saving therapy for ES and should not be used only as a bailout therapy. Interestingly, no significant benefit of RFCA with respect to composite of all-cause death, electrical storm recurrence and ventricular tachycardia/ventricular fibrillation recurrence was observed in the group treated with RFCA, compared with those in the remaining population. We hypothesise that the observed reduction of all-cause mortality in the RFCA group (despite lack of statistical significance in recurrence rates) was due to trigger and substrate modification caused by ablation. This modification in turn might have led to different arrhythmias (with more benign characteristics: slower and with lower burden). We can speculate that tissue oedema, which is created during ablation, could render a new reentrant circuit in the surrounding myocardium responsible for creation of new non-clinical VT (e.g. with lower cycle and burden). Indeed, our study has shown a high rate of early VA recurrence after RFCA procedure only within the 30 days after procedure compared with the conservative group (7 [25%] vs. 5 [12%], *p* = 0.16, respectively). On the other hand, if blanking periods were used (30 days), the recurrence rate was 46.4% in RFCA vs. 75% in non-ablated group; *p* = 0.02.

Our strong feeling has been based on the assumption that not every early recurrence will lead to late recurrence, and as such, it does not necessarily represent treatment failure. Lack of late recurrences in the ablated group, but their presence in the conservative group, might be responsible for survival benefit (what is also reflected by progressively more divergent with time Kaplan–Meier curves). However, this is only a hypothesis, which has to be confirmed in further studies.

Our concept is supported by an important recent study by Muser et al. [19]. The authors compared the long-term outcomes after VT ablation for ES in patients with non-ischemic dilated cardiomyopathy and ischemic cardiomyopathy. Compared to our results, the authors reported a lower incidence of VT recurrence (33%) in median follow-up 45 months. This difference may be related to the fact that in contrast to our study, they also included patients who underwent endo-epicardial RFCA (22%). Overall, this outcome may favourably affect long-term outcomes of RFCA. Although VT recurred in 33% patients after the ablation, a substantial reduction in VT burden was observed in most of these cases. Moreover, the majority of patients (69%) with VT recurrence had only isolated (≤ 3 VT) episodes during the 6 months after the RFCA procedure.

The open question remains, what is the rate of clinical to non-clinical arrhythmias among patients with recurrences and what is the impact of non-clinical VT or of early VA recurrence on clinical outcome?

This issue is even more interesting because, in contrast to our study, Sapp et al. recently reported a significantly lower rate of composite of death as the primary outcome, three or more episodes of ventricular tachycardia within 24 h (ventricular tachycardia storm) or appropriate ICD shock (59.1%) in groups of patients with ventricular tachycardia, who were randomly assigned to receive CA with continuation of baseline antiarrhythmic medication vs. 68.5% of those in the escalated pharmacological therapy group, *p* = 0.04 [20]. There was also no significant difference in mortality between the groups [20]. Their results may be explained by several reasons. The authors only conducted their analysis with a follow-up period of 27.9 ± 17.1 months in the group of patients with ischaemic cardiomyopathy after myocardial infarction and implanted with ICD, and who had experienced an episode of VT within the 6 months before enrolment. During follow-up, they analysed only the VT episodes and appropriate ICD shock after the 30-day treatment period. There is also lack of data on the percentage of previous RFCA and ES, which may reflect on their outcomes. Notably and surprising is the fact that our study, based on multivariable Cox regression analysis, provides the evidence that the presence of CRT-D is an independent predictor of all-cause mortality in patients with electrical storm. We suppose that this may be attributed to the fact that in contrast to ICD recipients, patients who initially demand the implantation of CRT-D are characterised by increased severity of heart failure, and may be much more exposed to the worsening of heart failure, resulting from multiple high-energy therapies within a short time period.

### Study limitation

The first important limitation is the relatively small sample size. However, because of the lack of data surrounding the efficiency of RFCA treatment in ES, our study contributes valuable information. Secondly, although the electrophysiologists who performed RFCA in our study were experienced in the procedure, we cannot rule out that specialised referral centres for endo- and epicardial RFCA could achieve better clinical outcomes. The third limitation is that we also cannot rule out that there might also be some preferences and intrinsic bias among practicing clinicians who perform CA procedures (e.g. preferring patients who are more likely to have a successful outcome for referring to the electrophysiology lab) which may have an influence on differences in the long-term treatment effect of ES in ablated and non-ablative group. The fourth and the most important limitation of our study was the retrospective analysis of short- and long-term outcomes in patients treated because of the occurrence of ES, and its retrospective comparison of RFCA treatment with non-RFCA therapy, not a controlled randomised trial comparing RFCA with other forms of treatment ES. Therefore, our study cannot unambiguously prove that RFCA is more effective than other therapies to enhance survival in patients with ES.

## Conclusions

By comparing long-time effects of ablation to other forms of electrical storm treatment among patients with heart failure and implanted ICD/CRT-D, who had a first episode of electrical storm, a statistically significant reduction in terms of total morality in the group treated with RFCA compared with those of the remaining population was observed.

## Electronic supplementary material

Table S1 (DOCX 89 kb)
