# Supplementary material for: Long-term outcome of catheter ablation and other form of therapy for electrical storm in patients with implantable cardioverter-defibrillators
Source: J Interv Card Electrophysiol. 2017 Oct 24;50(3):227–34. doi: 10.1007/s10840-017-0291-1 (PMC5729196; doi:10.1007/s10840-017-0291-1)
Supplement: Supplementary file 1 — (DOCX 89 kb) [file 10840_2017_291_MOESM1_ESM.docx]

|  | |  | | | Model 1 (p<0.05) | | | Model 2 (p<0.1) | | |
| --- | --- | --- | --- | --- | --- | --- | --- | --- | --- | --- |
| variable | | Univariate analysis | | | Multivariate analysis | | | Multivariate analysis | | |
|  |  | Hazard ratio | 95% confidence interval | p | Hazard ratio | 95% confidence interval | p-value | Hazard ratio | 95% confidence interval | p |
| Age |  | 1.03 | 0.98-1.08 | 0.20 |  |  |  |  |  |  |
| Gender |  | 1.99 | 0.58-6.81 | 0.27 |  |  |  |  |  |  |
| BMI (kg/m^2^) |  | 0.99 | 0.87-1.13 | 0.93 |  |  |  |  |  |  |
| NYHA class |  | 2.00 | 0.96-4.16 | 0.06 |  |  |  | 1.48 | 0.49-4.49 | 0.49 |
| LVEF (%) |  | 0.95 | 0.89-1.02 | 0.16 |  |  |  |  |  |  |
| MVR-severe |  | 5.34 | 1.19-23.95 | 0.03* | 1.17 | 0.14-9.68 | 0.89 | 1.55 | 0.15-15.88 | 0.71 |
| Implantation of ICD/CRTD in secondary prevention of SCA |  | 0.96 | 0.40-2.33 | 0.39 |  |  |  |  |  |  |
| During the storm, the presence of an implanted CRT-D |  | 5.37 | 2.10-13.74 | <0.001* | 7.99 | 2.35-27.16 | 0.001* | 6.83 | 1.93-24.24 | 0.003* |
| Ischemic cardiomyopathy |  | 0.95 | 0.32-2.85 | 0.93 |  |  |  |  |  |  |
| Previous myocardial infarction treated conservatively |  | 0.71 | 0.30-1.72 | 0.45 |  |  |  |  |  |  |
| Complete revascularisation after angiography |  | 0.71 | 0.29-1.71 | 0.44 |  |  |  |  |  |  |
| Diabetes type 2 |  | 4.05 | 1.62-10.17 | <0.01* | 2.76 | 0.90-8.39 | 0.07 | 3.40 | 0.98-11.86 | 0.05 |
| Chronic kidney disease |  | 2.16 | 0.89-5.24 | 0.09 |  |  |  | 0.40 | 0.09-1.84 | 0.24 |
| Stroke/ Transient ischaemic attack |  | 3.77 | 1.55-9.19 | 0.005* | 4.78 | 1.43-15.93 | 0.01* | 9.86 | 1.71-56.65 | 0.01* |
| Chronic obstructive pulmonary disease |  | 1.36 | 0.40-4.63 | 0.63 |  |  |  |  |  |  |
| Atrial fibrillation |  | 0.85 | 0.39-2.09 | 0.73 |  |  |  |  |  |  |
| Hematocrit level (%) |  | 0.87 | 0.77-0.98 | O.02* | 0.88 | 0.76-1.03 | 0.10 | 0.84 | 0.72-0.99 | 0.04* |
| C reactive protein (CRP) level (mg/l) |  | 1.03 | 1.01-1.104 | 0.001* | 1.01 | 0.99-1.04 | 0.20 | 1.02 | 0.99-1.05 | 0.13 |
| Creatynine level (umol/l) |  | 1.01 | 0.99-1.02 | 0.08 |  |  |  |  |  |  |
| GFR (ml/min/1.73m2) |  | 0.99 | 0.96-1.01 | 0.23 |  |  |  |  |  |  |
| NT-proBNP level (pg/ml) |  | 1.01 | 1.01-1.02 | <0.001* | 1.01 | 0.99-1.01 | 0.31 | 1.01 | 0.99-1.01 | 0.58 |
| Any potentially reversible cause of ES: |  | 2.78 | 1.14-6.79 | 0.03* | 1.92 | 0.57-6.50 | 0.29 | 0.32 | 0.01-92.0 | 0.69 |
|  |  |  |  |  |  |  |  |  |  |  |
|  | Acute coronary syndrome | 1.65 | 0.38-7.21 | 0.51 |  |  |  |  |  |  |
|  | Significant stenosis of the coronary artery requiring intervention | 2.24 | 0.92-5.44 | 0.07 |  |  |  | 3.55 | 0.02-826.0 | 0.65 |
|  | Infective endocarditis | 5.98 | 1.32-27.15 | 0.02* | 0.28 | 0.01-13.18 | 0.51 | 0.96 | 0.01-83.94 | 0.87 |
|  | Electrolyte imbalances | 6.31 | 0.77-51.89 | 0.09 |  |  |  | 7.99 | 0.23-2387.69 | 0.47 |
| Primary ablation |  | 0.34 | 0.11-1.01 | 0.05 |  |  |  | 0.47 | 0.11-1.88 | 0.29 |
| VT recurrence |  | 0.61 | 0.25-1.50 | 0.28 |  |  |  |  |  |  |
| ES recurrence |  | 0.92 | 0.37-2.25 | 0.85 |  |  |  |  |  |  |

**Table S1.** Univariate and multivariate overall survival analysis by the Cox proportional hazards model.

BMI; Body Mass Index, CRT-D; Cardiac Resynchronization Therapy Defibrillator, ES; Electrical Storm, GFR; Glomerular Filtration Rate, ICD; Implantable Cardioverter-Defibrillator, LVEF; Left Ventricular Ejection Fraction, MI; Myocardial Infarction MVR; Mitral Valve Regurgitation, NT-proBNP; N-terminal prohormone of brain natriuretic peptide, NYHA class; New York Heart Association class, SCA; Sudden Cardiac Arrest, VF; Ventricular Tachycardia, *p<0.05
